# Cardiovascular risk factors modulate the effect of brain imaging-derived phenotypes on ischaemic stroke risk

**DOI:** 10.1093/braincomms/fcaf183

**Published:** 2025-05-13

**Authors:** Yuan-yuan Liang, Meng-jie Li, Dong-rui Ma, Meng-nan Guo, Xiao-yan Hao, Shuang-jie Li, Chun-yan Zuo, Chen-wei Hao, Zhi-yun Wang, Yan-mei Feng, Chenyuan Mao, Chan Zhang, Bo Song, Yuming Xu, Changhe Shi

**Affiliations:** Department of Neurology, The First Affiliated Hospital of Zhengzhou University, Zhengzhou University, Zhengzhou, Henan 450000, China; Department of Neurology, The First Affiliated Hospital of Zhengzhou University, Zhengzhou University, Zhengzhou, Henan 450000, China; Department of Neurology, Academy of Medical Sciences of Zhengzhou University, Zhengzhou, Henan 450000, China; Department of Neurology, The First Affiliated Hospital of Zhengzhou University, Zhengzhou University, Zhengzhou, Henan 450000, China; Department of Neurology, The First Affiliated Hospital of Zhengzhou University, Zhengzhou University, Zhengzhou, Henan 450000, China; Department of Neurology, The First Affiliated Hospital of Zhengzhou University, Zhengzhou University, Zhengzhou, Henan 450000, China; Department of Neurology, Academy of Medical Sciences of Zhengzhou University, Zhengzhou, Henan 450000, China; Department of Neurology, The First Affiliated Hospital of Zhengzhou University, Zhengzhou University, Zhengzhou, Henan 450000, China; Department of Neurology, The First Affiliated Hospital of Zhengzhou University, Zhengzhou University, Zhengzhou, Henan 450000, China; Department of Neurology, The First Affiliated Hospital of Zhengzhou University, Zhengzhou University, Zhengzhou, Henan 450000, China; Department of Neurology, The First Affiliated Hospital of Zhengzhou University, Zhengzhou University, Zhengzhou, Henan 450000, China; Department of Neurology, The First Affiliated Hospital of Zhengzhou University, Zhengzhou University, Zhengzhou, Henan 450000, China; Department of Neurology, The First Affiliated Hospital of Zhengzhou University, Zhengzhou University, Zhengzhou, Henan 450000, China; Department of Neurology, The First Affiliated Hospital of Zhengzhou University, Zhengzhou University, Zhengzhou, Henan 450000, China; Department of Neurology, The First Affiliated Hospital of Zhengzhou University, Zhengzhou University, Zhengzhou, Henan 450000, China; NHC Key Laboratory of Prevention and treatment of Cerebrovascular Diseases, The First Affiliated Hospital of Zhengzhou University, Zhengzhou University, Zhengzhou, Henan 450000, China; Henan Key Laboratory of Cerebrovascular Diseases, The First Affiliated Hospital of Zhengzhou University, Zhengzhou University, Zhengzhou, Henan 450000, China; Institute of Neuroscience, Zhengzhou University, Zhengzhou, Henan 450000, China; Department of Neurology, The First Affiliated Hospital of Zhengzhou University, Zhengzhou University, Zhengzhou, Henan 450000, China; NHC Key Laboratory of Prevention and treatment of Cerebrovascular Diseases, The First Affiliated Hospital of Zhengzhou University, Zhengzhou University, Zhengzhou, Henan 450000, China; Henan Key Laboratory of Cerebrovascular Diseases, The First Affiliated Hospital of Zhengzhou University, Zhengzhou University, Zhengzhou, Henan 450000, China; Institute of Neuroscience, Zhengzhou University, Zhengzhou, Henan 450000, China; Department of Neurology, The First Affiliated Hospital of Zhengzhou University, Zhengzhou University, Zhengzhou, Henan 450000, China; NHC Key Laboratory of Prevention and treatment of Cerebrovascular Diseases, The First Affiliated Hospital of Zhengzhou University, Zhengzhou University, Zhengzhou, Henan 450000, China; Henan Key Laboratory of Cerebrovascular Diseases, The First Affiliated Hospital of Zhengzhou University, Zhengzhou University, Zhengzhou, Henan 450000, China; Institute of Neuroscience, Zhengzhou University, Zhengzhou, Henan 450000, China

**Keywords:** cardiovascular risk factors, brain imaging-derived phenotypes, ischaemic stroke, Mendelian randomization

## Abstract

Studies have shown that cardiovascular risk factors are closely related to the occurrence of stroke, especially ischaemic stroke, as they can lead to changes in brain structure and function. However, the role of cardiovascular risk factors—induced changes in brain structure and function in the development of ischaemic stroke has not been studied. The aim of this study is thus to explore the causal association among cardiovascular risk factors, brain phenotypes and ischaemic stroke by assessing Mendelian randomization. We used univariate Mendelian randomization to sequentially investigate the causal effects of the 12 most common cardiovascular risk factors on brain structure and 3935 brain imaging-derived phenotypes in the development of ischaemic stroke. We also examined the mediating effect of brain structure on blood pressure—induced ischaemic stroke using a multivariable Mendelian randomization test. We tested the reliability of our results using the Steiger test, heterogeneity test, horizontal pleiotropy test and leave-one-out method. We found that 8 of the 12 examined cardiovascular risk factors were associated with 538 brain imaging-derived phenotypes, and 9 of the 12 cardiovascular risk factors were associated with IS. The main cardiovascular risk factors associated with brain imaging-derived phenotypes and ischaemic stroke was blood pressure (systolic and diastolic), which can affect the occurrence of ischaemic stroke through 6 types of brain imaging-derived phenotypes. However, extrapolation of our findings to other ethnic groups is challenging, and the possibility of reverse causality cannot be completely ruled out. This study identifies the role of cardiovascular risk factors, especially blood pressure, in affecting brain structure and ischaemic stroke risk. The findings assist in early risk detection and enhance stroke prevention strategies, also hinting at non-vascular factors’ involvement.

## Teaser

Liang *et al*. report that cardiovascular risk factors significantly influence brain structure and function, with blood pressure specifically impacting six brain imaging-derived phenotypes linked to ischaemic stroke. This study underscores potential non-vascular mechanisms in stroke pathogenesis and highlights early identification and prevention strategies for high-risk individuals.

## Graphical Abstract

**Graphical Abstract:**
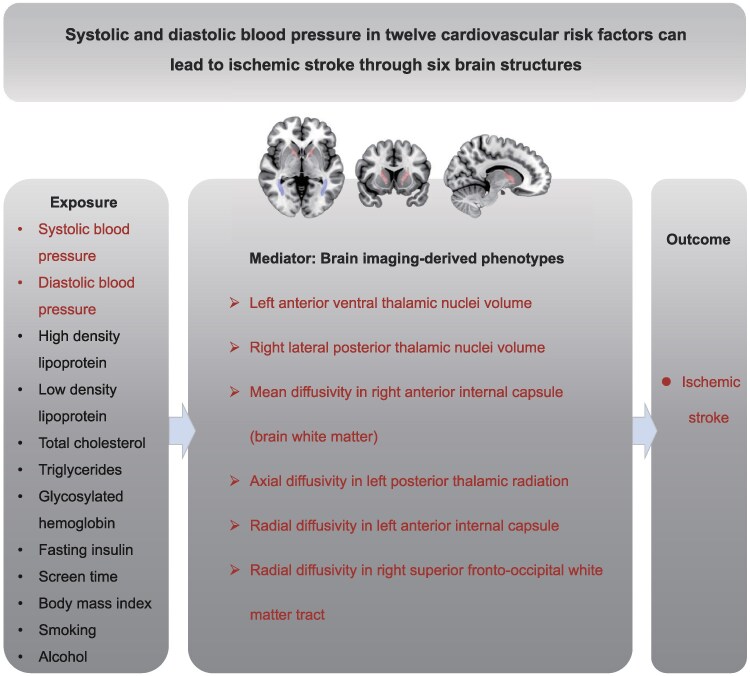

## Introduction

Stroke stands as a leading contributor to both disability and mortality,^1,2^ posing a significant challenge within the medical community. Numerous observational and prospective studies have elucidated that cardiovascular and cerebrovascular risk factors, including metabolic syndrome (hypertension, diabetes, hyperlipidaemia, obesity, etc.) and unhealthy lifestyle choices (smoking, lack of exercise, etc.), serve as primary aetiological agents for ischaemic stroke (IS).^3-5^ Recent investigations further substantiate that a genetic predisposition to elevated systolic and diastolic blood pressure (SBP and DBP, respectively) within the context of metabolic syndrome is significantly correlated with IS risk. Additionally, triglyceride levels and glycaemic indices not only enhance risk stratification but are also significantly associated with adverse outcomes in IS.^4,6,7^

Previous studies have suggested that cardiovascular risk factors (CRF) increase the risk of IS, mainly by damaging intracranial and extracranial blood vessels. In recent years, increasing evidence has shown that in addition to directly causing vascular diseases, metabolic syndrome and unhealthy lifestyle may have important effects on brain structure and function before IS occurs, including progressive changes in cortical thickness.^8,9^ Notably, extensive grey matter structural changes in the cortical and subcortical regions, as well as changes in the integrity of white matter fibre tracts, due to, for instance, higher body mass index (BMI) in middle age, lower likelihood of beta-amyloid positivity and larger mean hippocampal volume have been measured using structural or diffusion MRI.^10^ These findings indicate that the association between CRF and IS may be mediated in part by changes in brain structure and characteristics. Brain structural and functional changes, which form a large group of identifiable brain imaging-derived phenotypes (IDPs), can be useful indicators to measure brain structural, functional and substantive impairments, but the causal nature and mediating mechanism of this specific association are not yet clear.

Observational studies have demonstrated a more established association between IS and associated CRF. However, while these studies have yielded compelling results, their ability to infer causality is limited due to confounding and reverse causation. Mendelian randomization (MR) can overcome some of these biases and avoid certain confounding factors.^11^ Because the environment does not alter patients’ underlying genetics, the use of MR in turn reduces any potential reverse causality bias.^12^ In stroke cases, the MRI characteristics of brain structure changes before stroke cannot be reliably measured due to the great impact of IS on brain structure and function, but because genetic variations related to brain structure cannot be changed by disease events, they can serve as reliable indicators of brain structure before stroke. Therefore, MR is a useful method for assessing putative causal relationships between CRF, brain structure and IS.

**Figure 1:**
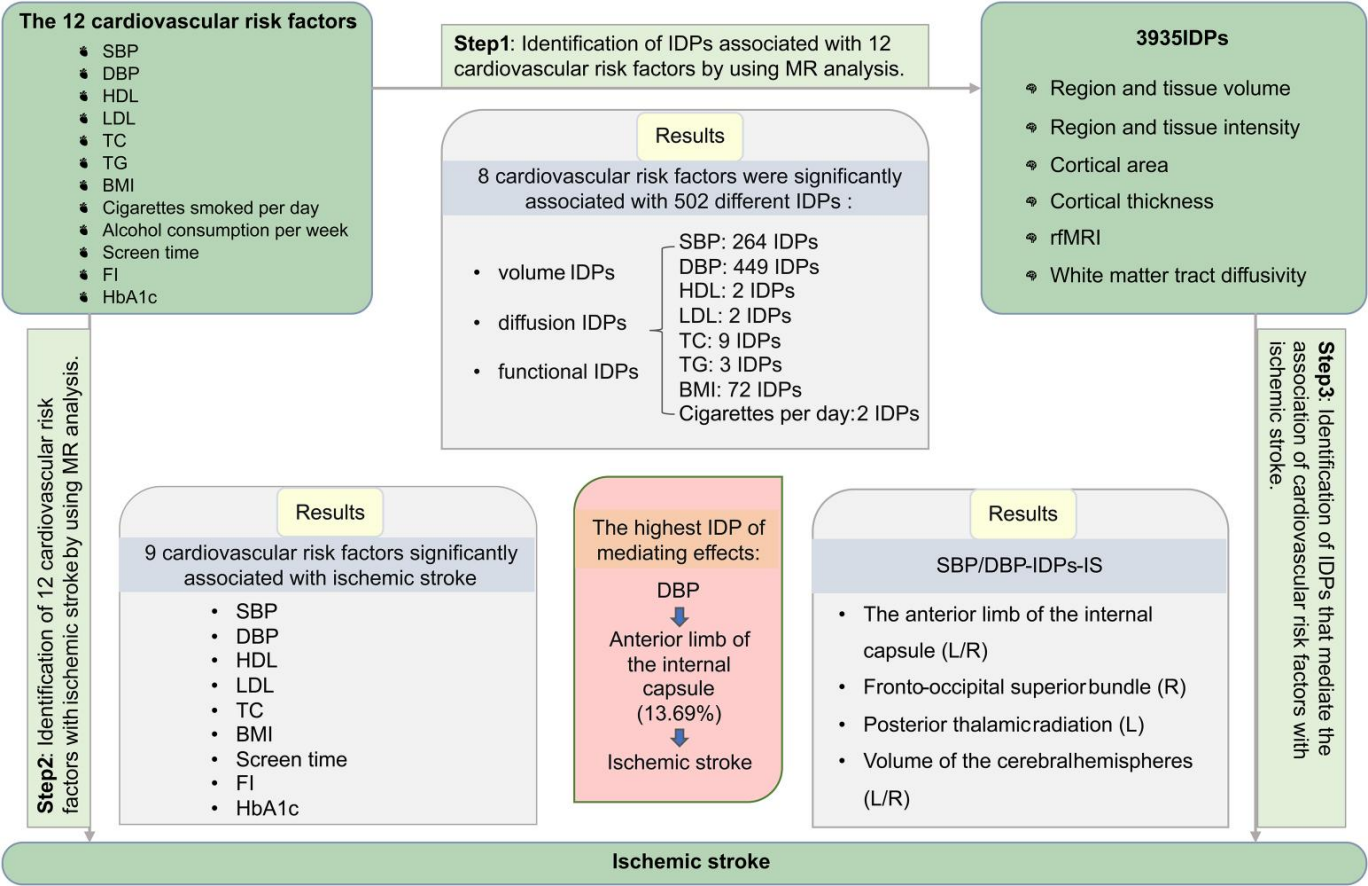
**Overview of this study.** There are three main steps in our study. First, we assessed the causal effects of 12 CRF measures on brain structure. Next, we used GWAS data to verify their impact on IS. Finally, we examined if brain structural changes mediate the effects of CRFs on IS risk. (DBP, diastolic blood pressure; SBP, systolic blood pressure; BMI, body mass index; TC, total cholesterol; TG, triglyceride; HDL, high-density lipoprotein; LDL, low-density lipoprotein; CpD, number of cigarettes smoked per day; FI, fasting insulin; HbA1c, glycosylated haemoglobin; L, left; R, right; rfMRI, resting-state functional MRI).

In this study, we aimed to find evidence that supports the existence of brain structural alterations in a causal pathway from CRF to IS. We selected 12 CRF that may be associated with IS from the most extensive studies to date, which fall under the categories BP (SBP and DBP), blood lipids (high-density lipoprotein (HDL), low-density lipoprotein (LDL), total cholesterol, triglycerides), blood glucose (fasting insulin, glycosylated haemoglobin) and lifestyle (BMI, daily smoking frequency, daily alcohol consumption, screen time). We also characterized brain structural changes using imaging markers (IDPs) of grey and white matter macro- and microstructures from structural or diffusion MRI. Our main objectives were to (i) assess whether the 12 CRF measures have a causal effect on brain macro- and/or microarchitecture; (ii) validate the causal role of the 12 CRF measures on the occurrence of IS using the latest or largest genome-wide association study (GWAS) data^13-15^ and (iii) to assess whether the macro- and/or microstructural IDP phenotypes found in response to the previous aim have a causal effect on the risk of IS, and whether the effect of the 12 CRF on stroke risk is mediated by changes in these structures (Fig. 1). This study has important clinical implications for exploring the brain phenotypes associated with CRF and the specific brain structures that mediate CRF and IS.

## Materials and methods

### Data source

In this study, 12 CRF were selected for analysis, all falling under the categories BP (SBP and DBP), blood lipids (HDL, LDL, total cholesterol, triglycerides), blood glucose (fasting insulin, glycosylated haemoglobin) or lifestyle (BMI, daily smoking frequency, daily alcohol consumption, screen time). The GWAS summary data for the above traits were all derived from studies of European patients, which included participants of both sexes, with sample sizes ranging from 45 734 to 757 601 people. These data can be found in a public database.

The pooled GWAS data for 3935 IDPs were obtained from the UK Biobank and included approximately 33 000 European patients. The IDPs formed 16 categories of brain structure and function,^16^ including brain area and tissue volume, cortical area, thickness, intensity and grey-white contrast. For specific information on IDPs, visit this website (https://open.win.ox.ac.uk/ukbiobank/big40/BIG40-IDPs_v4/IDPs.html).

GWAS summary data for IS were derived from 29 population-based cohorts or biobank studies and 25 clinic-based case-control studies, which included 66.7% European participants.^17^ We used European IS phenotypes as genetic estimates of the effect of the selected single nucleotide polymorphisms (SNPs) on stroke, with a sample size of 1 308 460 (73 652 European ancestry cases, 1 234 808 European ancestry controls). Relevant data can be accessed via this website (https://www.ebi.ac.uk/gwas/). While all the above data came from different organizations, after manual screening, there was no sample overlap.

### Selection of genetic tools

We selected independent SNPs for the corresponding GWAS data upon determining the 12 CRF or brain phenotypes as exposures, as based on the linkage disequilibrium clustering of *P* < 5E-8, *R*^2^ < 0.1 within a 500-kb window, using 1000 European ancestry genomic data as the reference dataset. This allowed us to obtain 60–1290 SNPs in CRF and 1–62 SNPs in IDPs for subsequent MR analysis.

### Statistical analysis

We used univariate MR analysis to assess the overall causal effect of (i) the 12 CRF on brain phenotypes, (ii) the effect of IS and (iii) the brain phenotypes associated with IS. When the number of SNPs was >1, we used inverse variance weighting MR with multiplicative random effects as the primary method of analysis, because it provided the most efficient combination of estimates of the specific variable ratios and accounted for heterogeneity in the causal estimates. When the number of SNPs was equal to 1, we used the Wald ratio method to explore the causal relationship between exposure and outcome. Multivariate Mendelian analysis was performed to adjust for the effects of both SBP and DBP on IDPs when BP indications had the most significant effects. Then, we corrected our results with Bonferroni method.

### Intermediary analysis

We used regression-based multivariate MR adjusted correlated brain phenotypes to estimate the direct effect of CRF on IS risk. The direct effect was then subtracted from the total causal effect derived in univariate MR to obtain the mediating effect of the relevant brain phenotype. The proportion of brain phenotypes in IS mediated by the corresponding CRF was estimated by dividing the mediating effect by the total effect.

### Sensitivity analysis

MR analysis was performed using TwoSampleMR software. To avoid using a reverse measure tool to detect the directionality of our results, we employed the Steiger test, an extension of two-sample MR, which detects variation with stronger associations with outcome than with exposure. At the same time, we applied Bonferroni correction to test the accuracy of our results. We used MR Egger, weighted median, simple mode and weighted mode as well to verify the robustness of the inverse variance weighting results in the univariate MR analysis. We then performed heterogeneity, level pleiotropy and leave-one-out tests. The MR analysis and TwoSample MR package were performed in R (version 4.2) using MR.

## Results

### Eight cardiovascular risk factors identified as associated with 538 imaging-derived phenotypes

**Figure 2:**
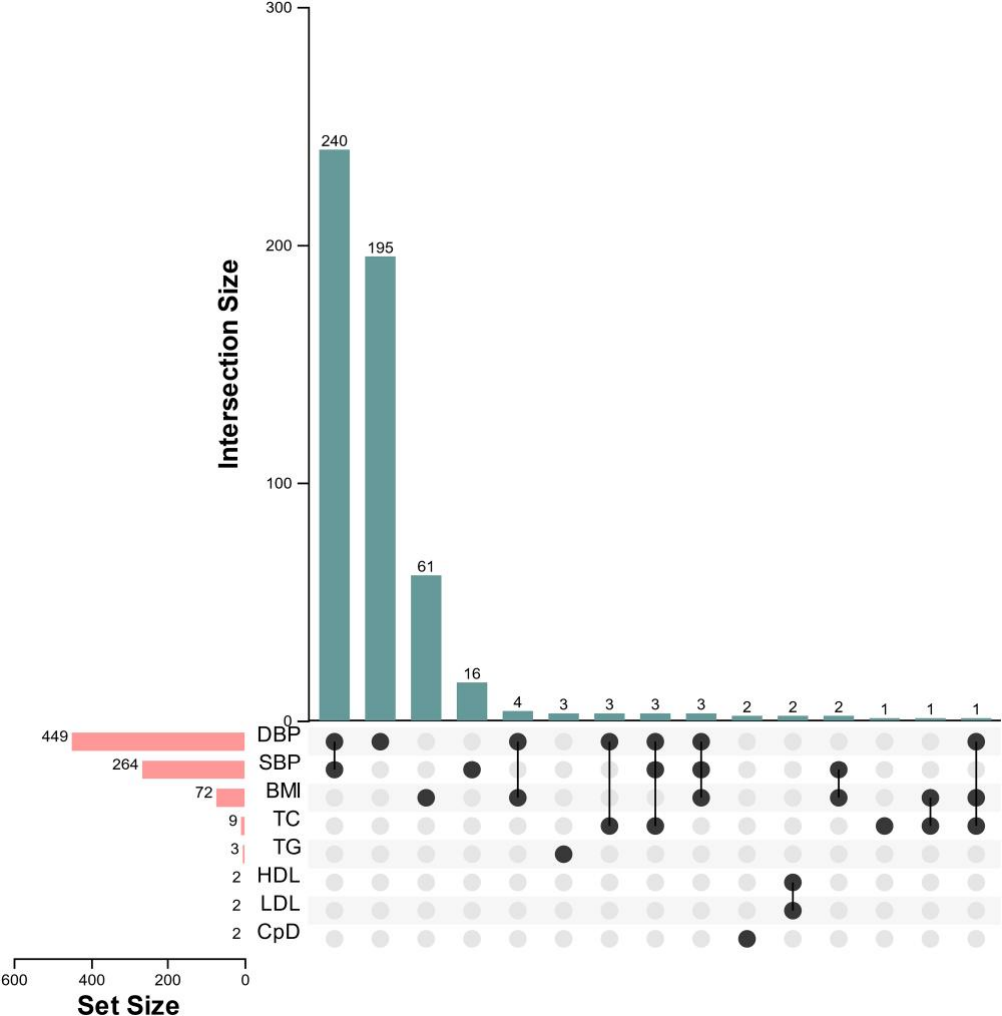
**Upset Venn diagram of the results from univariate Mendelian analysis of 12 CRF and IDP.** The rectangle on the left shows that DBP-related IDPs have the largest number of 449 IDPs. There were 195 IDPs affected by DBP alone, and 240 IDPs affected by both SBP and DBP. (DBP, diastolic blood pressure; SBP, systolic blood pressure; BMI, body mass index; TC, total cholesterol; TG, triglyceride; HDL, high-density lipoprotein; LDL, low-density lipoprotein; CpD, cigarettes per day).

Because CRF has been associated with brain structure and function in observational analyses, we used univariate MR analysis to further explore brain phenotypes that may be causally associated with CRF. For deeper review of the causal association between CRF and IDPs at the genetic level, we performed univariate MR analysis of 12 CRF as exposures and 3935 IDPs as outcomes, finding that 8 CRF were associated with 538 IDPs after Bonferroni correction (Fig. 2). The CRF measures BMI, daily smoking frequency, SBP, DBP, HDL and LDL, total cholesterol and triglycerides were associated with 72, 2, 264, 449, 2, 2, 9 and 3 IDPs, respectively, suggesting that different CRF may have different effects on IDPs (Supplementary Table 1). About 7.13% of the results had horizontal pleiotropy (Supplementary Table 9), but most of the results were heterogeneous (Supplementary Table 10). All Steiger tests were TRUE (Supplementary Table 8). However, different CRF may also have a causal effect on the same IDPs. The main types of IDPs associated with CRF were diffusion-weighted, volumetric and resting-state functional IDPs.

Notably, the largest number of IDPs were affected by BP indications and the most significant correlation was with white matter tract diffusion capacity (264 and 449 IDPs affected by SBP and DBP, respectively). When both levels were increased, there was a negative effect on the white matter tract, especially its diffusion and related parameters, fractional anisotropy (FA) and isotropic volume fraction. Further examination revealed that 246 IDPs were affected by both SBP and DBP. To avoid their confounding, we performed multivariate MR analysis of these factors with 538 IDPs (Supplementary Table 5). The results showed that SBP was significantly associated with 234 IDPs and DBP with 444 IDPs. Compared with univariate Mendelian analysis, the number of IDPs related to BP decreased slightly. Importantly, for any leave-one-out inverse variance weighting MR analysis about 90% of all the above correlations remained significant at nominal *P* < 0.05.

In addition, we observed that BMI and lipids were most significantly associated with IDPs after the BP analysis. Univariate MR analysis showed that BMI was negatively correlated with resting-state functional IDPs, and positively correlated with most diffusion-weighted and volumetric IDPs. The effect of blood lipids on the development of IDPs was also different. Total cholesterol affected a relatively large number of IDPs, mainly white matter tract diffusion capacity, especially in the white matter tracts of the corona radiata. HDL, LDL and triglyceride also had negative effects on cerebral cortex and volume.

Regarding lifestyle, only daily smoking frequency had a potential causal effect on IDPs. Daily smoking had a negative effect on the volume of the right hemisphere and the mean diffusion tensor of the pontine crossing tract. As the frequency of smoking increased, the decussating tracts of the pons, which are involved in motor control and sensory transmission processes within the brainstem, may have been affected. In conclusion, a number of CRF are significantly associated with IDPs, with BP having the most extensive effect, mainly on white matter tract diffusion capacity.

### Identification of nine cardiovascular risk factors with potential causal effects on ischaemic stroke

**Figure 3:**
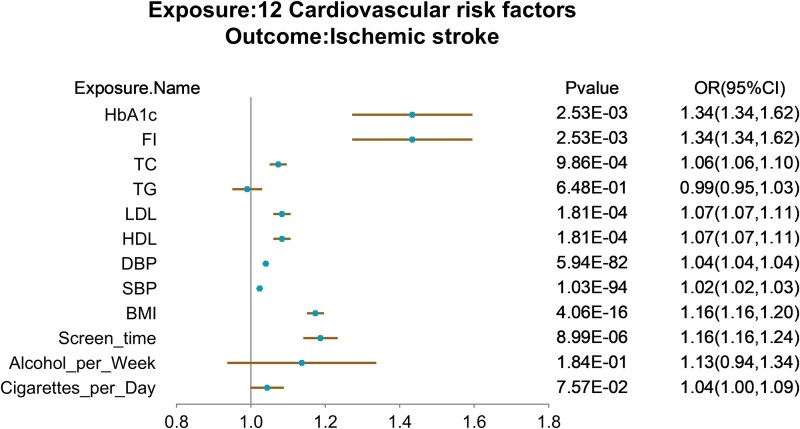
**Forest plot of univariate Mendelian randomization using the inverse variance weighted method for 12 cardiovascular risk factors in relation to IS.** The results indicated that systolic and diastolic BP had the most significant causal effects. The *x*-axis is the OR (95% CI) and the *y*-axis is the different exposure. OR (95% CI), odds ratio (95% confidence interval).

Next, we further explored the causal association between the 12 CRF and IS at the genetic level. We used the 12 CRF as exposures and IS as an outcome for univariate MR analysis with Bonferroni correction (Supplementary Table 2). Nine CRF were significantly associated with IS, which were SBP, DBP, HDL, LDL, total cholesterol, fasting insulin, glycosylated haemoglobin, BMI and screen time (Fig. 3). Interestingly, SBP (*P* = 1.03E-94) and DBP (*P* = 5.94E-82) had the most significant causal associations with IS, as validated by sensitivity analyses. In addition, blood glucose (*P* = 2.53E-03), glycosylated haemoglobin (*P* = 2.53E-03), BMI (*P* = 4.06E-16) and screen time (*P* = 8.99E-06) were all IS risk factors. However, after inverse variance weighting analysis, we found no potential causal relationship between lipid triglycerides and IS. Overall, there were significant associations between multiple CRF and IS, with the most significant causal association between BP and IS.

### Six brain phenotypes associated with cardiovascular risk factors and significantly associated with ischaemic stroke

**Figure 4:**
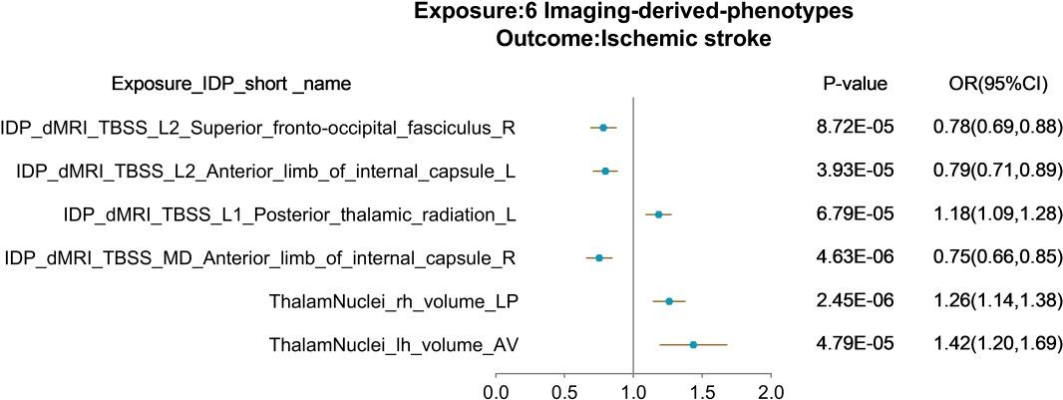
**Forest plot of univariate Mendelian randomization using the inverse variance weighted method for six brain imaging-derived phenotypes in relation to IS.** The anterior limb of the internal capsule and the fronto-occipital tract exhibit negative effects on IS, while the posterior thalamic radiation and brain volume exhibit positive effects. The *x*-axis is the OR (95% CI) and the *y*-axis is the different exposure. (OR (95%CI), odds ratio (95% confidence interval); IDP, imaging derived phenotypes; dMRI, diffusion MRI; TBSS, tract-based spatial statistics; L1, axial diffusivity; L2, second Eigenvalue; MD, mean diffusivity; FA, fractional anisotropy; LP, lateral posterior nucleus; AV, anteroventral nucleus; lh/rh: left hemisphere/right hemisphere).

To further explore the causal association between IDPs, CRF and IS, we placed the CRF associated with IS in the context of brain phenotypes also associated with CRF. Nine CRF associated with IS were further screened, and in these results, a total of 538 IDPs were associated with 8 CRF. After screening for CRF that were associated with both IS and IDPs, we obtained 502 associated IDPs. We also performed univariate MR analysis (Supplementary Table 3) and Bonferroni correction (0.05/502) using 502 IDPs and IS. From this analysis, we found six IDPs, mainly focused on regional tissue volume and white matter tract diffusion capacity, to be significantly associated with IS: left and right internal capsule anterior limb; right superior fronto-occipital tract; left posterior thalamic radiation; and left and right cerebral hemisphere volume (Fig. 4, Supplementary Table 4). Interestingly, all of these IDPs were significantly associated with BP, but not with other CRF. In conclusion, we found that 6 IDPs were significantly associated with IS, all of which were significantly associated with BP. We thus speculate that these IDPs may play a mediating role in specifically hypertensive IS.

### The mediating effect of imaging-derived phenotypes in the blood pressure-imaging-derived phenotypes-ischaemic stroke axis

**Figure 5:**
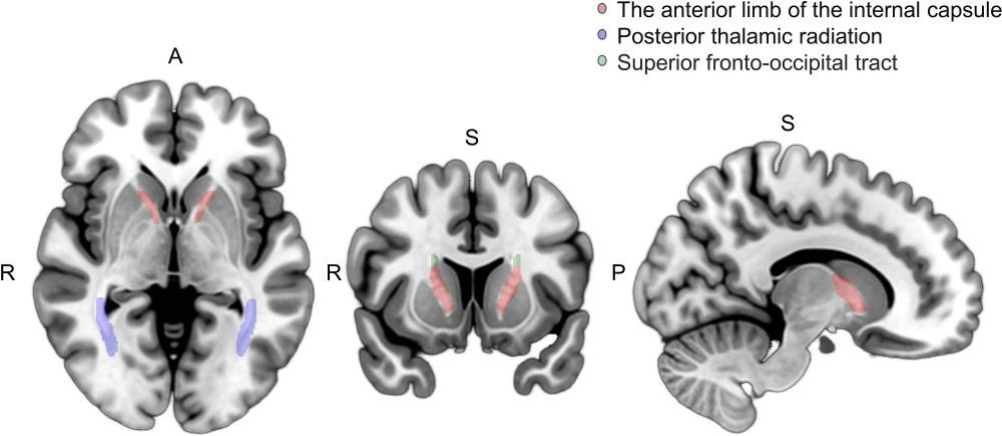
**Pattern of brain structure.** Brain structural patterns of white matter associated with cardiovascular risk factors and a significant effect on IS. (R, right; A, anterior; S, superior; P, posterior).

To further explore the mediating effect of IDPs on hypertension-induced IS, we incorporated six IDPs associated with IS into multivariate Mendelian analysis with SBP and DBP, respectively (Supplementary Table 6). The results showed that the effect size directions of three IDPs in the SBP analysis were inconsistent, so we did not consider their mediating effects for that analysis. Excitingly, after adjustment of the right anterior limb of the internal capsule, the direct effect of SBP on IS was beta 0.02 (*P* = 4.68E-82), and the direct effect of DBP on IS was beta 0.03 (*P* = 5.82E-46); after adjustment of the left anterior limb of the internal capsule, the direct effect of SBP on IS was beta 0.02 (*P* = 1.34E-82), and the direct effect of DBP on IS was beta 0.03 (*P* = 1.04E-46); after adjustment of the right superior fronto-occipital fasciculus, the direct effect of SBP on IS was beta 0.02 (*P* = 3.60E-72), and the direct effect of DBP on IS was beta 0.03 (*P* = 1.81E-51). Among them, DBP-left internal capsule forelimb-IS had the largest mediating effect at 13.69%, followed by DBP-right internal capsule forelimb-IS at 13.31%. This was followed by DBP-right superior fronto-occipital fasciculus-IS (10.32%), SBP-right anterior internal capsule-IS (7.64%), SBP-left anterior internal capsule-IS (6.02%), SBP-right superior fronto-occipital fasciculus-IS (4.80%), DBP-left hemisphere volume-IS (2.46%) and DBP-left posterior thalamic radiation-IS (2.28%) (Fig. 5, Supplementary Table 7). The anterior limb of the internal capsule, which had the largest proportion of mediating effects, may therefore become the focus of attention in the occurrence of hypertensive IS.

## Discussion

In this study, we performed a two-sample univariate MR analysis using a large GWAS dataset, which comprehensively revealed the causal relationship between 12 common CRF, 3935 brain phenotypes and IS at the genetic level. We identified 538 IDPs associated with CRF and a causal link between 9 CRF and IS. Finally, Mendelian mediation analysis revealed that hypertension could affect the occurrence of IS through six brain phenotypes (left and right internal capsule anterior limbs, left and right hemispheric specific volume, right superior frontal occipital tract and left posterior thalamic radiation). We now further discuss this causal relationship between CRF, IDPs and IS.

Of the CRF most associated with the occurrence of IS, BP had the most significant association with IS. Moreover, we found that multiple IDPs were significantly associated with CRF and IS. Previous cohort studies have confirmed the association between hypertension and IDPs.^18-21^ Likewise, we found a negative relationship between BP and a number of IDPs, particularly in the majority of structures associated with white matter tracts, such as the anterior limb of the internal capsule, external capsule, corona radiata, posterior thalamic tract, superior longitudinal fasciculus and total volume of white matter hyperintensities. Similarly, multiple case-control studies have revealed that IS is closely associated with several of these same IDPs, including the anterior and posterior limbs of the internal capsule, the superior longitudinal fasciculus and the posterior thalamic radiation.^22,23^

It is worth noting that of all CRF, BP showed the strongest association with both IDPs and IS, suggesting that BP may be a predisposing factor for the occurrence of IS. However, previous studies have found that before the occurrence of the IS, IDPs themselves are affected by many risk factors, such as metabolic syndrome and unhealthy lifestyle.^10^ This suggests that a change in IDPs may be a proxy for risk factors and mediate IS occurrence. Specifically, in addition to the traditional vascular factors that can mediate the impact of BP on IS, non-vascular factors can also affect BP and the occurrence of IS to a certain extent.

Mendelian mediating effect analysis revealed that six IDPs mediated the occurrence of IS as caused by CRF, of which only BP was significantly associated with these IDPs. This further defends that change in IDPs plays an important role in the occurrence of hypertensive-related IS. Interestingly, in the mediation analysis, the anterior limb of the internal capsule had the highest proportion of mediating effects in hypertensive IS, suggesting that structural abnormalities in this region play an important role in hypertensive-related IS and can be used as a predictor of its occurrence. The anterior limb of the internal capsule serves as a critical hub for the transmission of motor and limbic circuit information,^24-26^ which is intricately linked to cognitive processes, emotional regulation, decision-making, resilience, and motor functionality.^27-30^ Existing research indicates that this region is richly vascularized,^31^ suggesting that hypertension could elevate vascular pressure, thereby potentially impacting both blood perfusion and functional integrity in this area.^32^ This results in decreased blood supply and nerve fibre damage in this region,^29,33^ in turn causing structural changes, which provides an explanation for the association between hypertension, vascular function and IDPs. Although other CRF beyond BP have significant causal associations with some IDPs and IS, this study did not reveal more evidence that IDPs mediate the occurrence of IS caused by those CRF.

Several studies have shown a close association between BMI and IDPs. For example, BMI can affect cerebral cortex and subcortex volume.^34,35^ In addition, studies have suggested that increased BMI is significantly associated with thinning of the left lateral occipital cortex and the right ventromedial prefrontal cortex.^34^ The results of this study differed from those of previous studies, revealing that increased BMI can instead cause volume changes in the inferior parietal cortex and the middle temporal region of the left cerebral hemisphere. The inferior parietal cortex, located in the upper posterior region of the brain, plays a significant role in spatial awareness and attention, while the middle temporal region, situated in the lateral aspect of the brain's temporal lobe, is primarily involved in semantic memory processing and language comprehension.^36,37^ Although our results did not show that BMI regulates changes in IDPs, thereby leading to IS, they did emphasize the role of BMI in neurological diseases, meaning BMI should still be a consideration in the study of neurological diseases.

Although lipids are a clear risk factor for IS, whether they are related to changes in brain structure and function is not clear. Research has shown that high cholesterol levels, especially high LDL levels, are associated with hippocampal volume shrinkage and structural abnormalities in other brain regions,^38^ as well as with cognitive decline and memory impairment.^39^ However, our results did not indicate that lipid-related indicators were associated with reduced brain volume, particularly hippocampal volume. Rather, this study revealed that there may be a causal relationship between HDL, LDL and the specific volume of the left, right hemispheres, corona radiata, which may be related to the different sources of data in our study. In addition, we found that there is a certain negative relationship between blood lipids and the corona radiata of the cerebral white matter tract. This region is a white matter structure composed of myelinated axonal fibrer that converge and diverge to connect the cerebral cortex with the brain stem, serving as a crucial conduit for both ascending sensory information and descending motor commands.^40,41^ These results suggest that lipid-lowering drugs such as statins may have a potentially protective effect on the white matter tract, especially on the thinking, perception and motor control functions involved in the corona radiata.

In this study, we found that both of the glucose-related CRF (fasting insulin and glycosylated haemoglobin) were significantly associated with IS, but there was no evidence of a potential causal association between them and IDPs. Previous studies have shown that blood glucose is associated with reduced brain grey matter volume,^42^ and upon diffusion tensor imaging, some patients with type 1 diabetes exhibit white matter microstructural defects, especially in some areas of the corona radiata. We speculate that these differences may be due to the insufficient power of evidence test caused by the different sample sizes of the GWAS, and the number of SNPs used in our study versus previous research.^42^

Smoking, drinking and screen time are related to brain structure,^43-45^ which is related to the destruction of nerve fibres, especially white matter abnormalities.^46,47^ However, this study revealed that daily smoking frequency was negatively correlated with right hemisphere volume and the pontine crossing tract, which has not been reported in previous studies. However, the lack of standardized lifestyle metrics in current research calls for more rigorous, uniform analyses to further explore the causal links between unhealthy lifestyle habits and IDPs.

Specifically, the following brain structures showed important changes: the volume of the anterior ventral in the left hemisphere and the volume of lateral posterior, in the right hemisphere, which were subdivided by the subcortical volume of thalamic nuclei, suggesting that these regions may be involved in neural pathways related to blood pressure regulation. Mean mean diffusivity (MD) in the anterior limb of the internal capsule (right), mean L2 in the anterior limb of the internal capsule (left), and mean L2 in the superior fronto-occipital tract (right), diffusivity measures derived from diffusionMRI data reflect the microstructural integrity of white matter pathways, changes in which may predict pathological changes in nerve fibres. Mean L1 in the posterior thalamic radiation (left), further highlights the involvement of important neural conduction pathways. These alterations in brain structure can be used as early biomarkers in patients at high risk of IS to help physicians in risk assessment and disease progression monitoring. Treatment is modified according to the characteristics of brain imaging, which may include more aggressive blood pressure management strategies or targeted neuroprotective therapies. These results provide further genetic support for the association between altered brain structure and IS. At present, it has been proven that changes in white matter hyperintensity detected by MRI technology are associated with an increased risk of cerebral small vessel disease. The changes of white matter hyperintensities have been used to predict cerebral small vessel disease and show good predictive efficiency. Suggesting the results as a potential risk prediction of IS in early biomarkers has huge potential. In addition, adjusting blood pressure or neuroprotective treatment according to different brain structural characteristics of patients may be helpful for the treatment of IS.

Our study found that increased MD in the anterior limb of the internal capsule (right) measured by fractional anisotropy (FA) skeleton based on diffusion MRI data was associated with elevated diastolic blood pressure and increased risk of IS, which are markers of microstructural damage or changes within the brain white matter pathways. This association suggests that MD has the potential to serve as a biomarker of IS risk, especially for patients with elevated diastolic blood pressure. Clinicians may consider incorporating diffusion MRI assessment into the routine evaluation of patients at high risk of IS. Incorporating FA and MD measures into risk assessment models could improve IS prediction accuracy, allowing for earlier and more targeted interventions. Identifying high MD values in high-risk patients may lead to more aggressive management of blood pressure and other modifiable stroke risk factors. Further research should be aimed at depicting the blood pressure and brain microstructure changes between the causal mechanism, and is likely to explore treatment goals to reduce these effects.

Although this study provides a comprehensive understanding of the causal association between CRF, IDPs and IS, the following limitations should be considered. First, our study mainly used the data of people from Europe. Therefore, it is challenging to extrapolate the results to other ethnic groups. Second, the number of subjects in the brain phenotypic GWAS is not large (approximately 33 000 people); therefore, the number of genetic instruments available for a particular IDP is relatively small or even lacking.^16^ In addition, although causal inference based on MR is not affected by confounding factors and reverse causality, and the possibility of reverse causality is small after careful selection of instrumental variables, it cannot be completely ruled out from our results. Third, IS is primarily a vascular disease, but brain phenotype data were missing for the intracranial and extracranial macrovascular phenotypes. Fourth, we selected from the total IS data and did not refine it to each subtype. Therefore, if we want to explore the relationship between CRF, brain phenotype and large artery stroke, small artery stroke and cardiac stroke, further research is needed. Finally, while the results of this study highlight the BP-IDPs-IS axis relationship, further epidemiological characterization is needed to verify our results’ reliability.^48^

## Conclusions

The present study provides the first comprehensive understanding of the causal relationship between CRF, brain phenotypes and IS via MR analysis, with particular emphasis on the BP-IDPS-IS axis and changes in brain structure and function as related to IS-associated CRF. These results can aid further understanding of the role of non-vascular factors in the pathogenesis of IS, the identification of populations at high risk of IS and the formulation of more effective stroke prevention and treatment strategies.

## Supplementary Material

fcaf183_Supplementary_Data

## Data Availability

GWAS summary data of 12 CRFs can be found in a public database (https://gwas.mrcieu.ac.uk/). The pooled GWAS data for 3935 IDPs were obtained from the UK Biobank (https://open.win.ox.ac.uk/ukbiobank/big40/).
